# Prosthetic and Biological Complications of Metal–Zirconia versus Metal–Acrylic Implant-Supported Screw-Retained Complete-Arch Prostheses: A Systematic Review and Meta-Analysis

**DOI:** 10.4317/jced.63882

**Published:** 2026-04-25

**Authors:** Dusan Marinkovic, Rodrigo Gutiérrez, Marcos Frey, Francisca Collao, Yasna Moreno, Claudio Miranda

**Affiliations:** 1 DDS. Professor, Postgraduate Program in Oral and Maxillofacial Implantology, School of Dentistry, Faculty of Medicine, Clínica Alemana–Universidad del Desarrollo, Santiago, Chile; 2 DDS, PhD Professor, Postgraduate Program in Oral and Maxillofacial Implantology, School of Dentistry, Faculty of Medicine, Clínica Alemana–Universidad del Desarrollo, Santiago, Chile; 3 DDS. Department Head and Faculty Member, Postgraduate Program in Oral and Maxillofacial Implantology, School of Dentistry, Faculty of Medicine, Clínica Alemana–Universidad del Desarrollo, Santiago, Chile

## Abstract

**Background:**

Full-arch implant-supported screw-retained prostheses are widely used for the rehabilitation of edentulous patients. Conventional metal-acrylic hybrid prostheses have demonstrated acceptable clinical outcomes, however, mechanical complications, prosthetic wear, and biological issues remain concerns. Monolithic zirconia prostheses with a metallic substructure have been proposed as an alternative, potentially improving long-term clinical performance.

**Material and Methods:**

A systematic review and meta-analysis were conducted following predefined eligibility criteria. A comprehensive search of CENTRAL, PubMed, Embase, and Epistemonikos databases was performed for studies published between 2000 and 2025. Non-randomized clinical studies with a minimum follow-up of 1 year comparing full-arch implant-supported screw-retained metal-zirconia prostheses with metal-acrylic prostheses were included. Fully monolithic zirconia restorations, partial fixed prostheses, and non-full-arch rehabilitations were excluded. Risk of bias was assessed using the ROBINS-I tool, and the certainty of evidence was evaluated according to the GRADE approach. Quantitative synthesis was conducted when appropriate.

**Results:**

Twelve studies fulfilled the inclusion criteria, with eleven included in the meta-analysis. Metal-zirconia prostheses showed significantly lower prosthetic wear (RR=0.29; 95% CI, 0.14-0.59) and a reduced risk of peri-implantitis (RR=0.67; 95% CI, 0.47-0.96). Marginal bone loss favored metal-zirconia prostheses (SMD=0.36). Trends toward improved prosthesis survival (RR = 1.05) and reduced prosthesis loss (RR = 0.09) were observed. No significant differences were found regarding implant survival, framework fractures, esthetics, or speech. Most studies exhibited moderate to critical risk of bias, mainly due to confounding factors.

**Conclusions:**

Within the limitations of predominantly non-randomized evidence, metal-zirconia implant-supported screw-retained complete-arch prostheses demonstrate a more favorable clinical profile than metal-acrylic prostheses, particularly in terms of prosthetic wear, peri-implantitis, and marginal bone loss. Although overall survival rates were comparable, trends toward improved prosthesis longevity were observed. Well-designed randomized clinical trials with long-term follow-up are required to confirm these findings.

## Introduction

Complete edentulism constitutes a complex clinical scenario with profound functional, esthetic, and psychosocial implications, substantially impacting patient quality of life and representing a significant public health concern due to its high prevalence in the elderly and association with systemic comorbidities. Oral implantology has emerged as the treatment modality of choice over conventional removable prostheses, providing more predictable, stable, and durable rehabilitations. Implant-Supported Screw-Retained Complete-Arch Prostheses (IS-CAPs), fixed intraorally yet retrievable for maintenance, optimize masticatory function, esthetics, and facial support while circumventing the biomechanical limitations inherent to conventional alveolar ridge-supported prostheses (1 , 2). IS-CAPs incorporate design features that render them particularly suitable for atrophic alveolar ridges: (a) they enable full-arch rehabilitations supported by only four to six implants; (b) they allow strategic implant angulation to maximize bone contact and avoid critical anatomical structures, reducing distal cantilevers and optimizing load distribution; (c) their arched configuration provides quadrangular anchorage to counteract occlusal forces and facilitate immediate loading when indicated; and (d) they minimize the need for extensive bone augmentation by efficiently utilizing residual bone.(3) IS-CAPs also confer reduced occlusal loading on soft tissues, enhanced esthetics through acrylic, resin, or zirconia veneering, improved patient comfort, and masticatory efficiency approaching that of natural dentition. Their retrievability allows for hygiene and prosthetic adjustments, achieving high survival rates for both prostheses (93-100%) and supporting implants (88-100%). However, reported limitations include food impaction, phonetic alterations, hygiene challenges, risk of mucositis and peri-implantitis, acrylic tooth fractures, mechanical misfits, marginal bone loss, and increased cost and technical complexity associated with CAD/CAM planning and multi-implant frameworks (2 , 4). Material selection is a critical determinant of IS-CAPs longevity, functionality, and esthetics. Metal-acrylic (MA) systems, while cost-effective and easily fabricated, are susceptible to long-term complications such as wear, fractures, and debonding, necessitating periodic maintenance (5). Metal-zirconia (MZ) IS-CAPs have been developed to address these limitations, providing superior wear resistance, color stability, and biocompatibility, thereby enabling more predictable and durable rehabilitations. The principal drawback remains the higher cost and technical complexity of fabrication associated with zirconia materials. The objective of this study is to conduct a systematic review and meta-analysis to evaluate the clinical performance of monolithic zirconia prostheses with a metallic framework compared to conventional metal-acrylic IS-CAPs, focusing on the incidence of prosthetic complications.

## Material and Methods

This systematic review and meta-analysis was conducted in full accordance with the Preferred Reporting Items for Systematic Reviews and Meta-Analyses (PRISMA) guidelines and was prospectively registered in the PROSPERO database (CRD420251090216) (6). The research question was structured according to the PICO framework as follows: Population (P): patients rehabilitated with implant-supported screw-retained complete-arch prosthesis; Intervention (I): monolithic zirconia IS-CAPs with a metallic substructure; Comparison (C): conventional metal-acrylic IS-CAPs; and Outcome (O): clinical performance in terms of prosthetic complications. Accordingly, the research question was formulated as follows: In patients rehabilitated with Implant-supported screw-retained complete-arch prosthesis, do monolithic zirconia prostheses with a metallic substructure demonstrate superior clinical performance, in terms of prosthetic complications, compared to conventional metal-acrylic hybrid prostheses? The inclusion and exclusion criteria were defined according to the PICO framework. Eligible studies comprised randomized controlled trials, non-randomized clinical trials, and prospective or retrospective cohort studies published in peer-reviewed journals. In vitro studies, case reports, case series, narrative reviews, previous systematic reviews, editorials, and expert opinions were excluded. The selected studies included patients rehabilitated with IS-CAPs who had completed both the surgical and prosthetic phases, and with a minimum clinical follow-up of one year. Studies involving patients without definitive prosthetic rehabilitation or with insufficient follow-up data were excluded. The intervention of interest consisted of implant-supported screw-retained complete-arch prostheses (IS-CAPs) fabricated from monolithic zirconia materials with a metallic substructure, excluding single crowns, partial fixed prostheses, removable prostheses, and overdentures. The intervention of interest was restricted to implant-supported screw-retained complete-arch prostheses supported by a full-arch metallic bar-type framework as the definitive substructure. Titanium abutments, ti-bases, intermediate inserts, or partial metallic components were not considered equivalent to a metallic substructure and were therefore excluded. The comparison group included conventional metal-acrylic IS-CAPs with a metallic substructure; studies comparing other types of rehabilitations were excluded. Only studies reporting prosthetic complications, including framework fracture, decementation, wear, replacement of teeth or screws, and acrylic or zirconia fractures, were included, while those lacking data on prosthetic complications or with unavailable outcome data were excluded. Publications from the year 2000 onward were considered, with no language restrictions applied. An exhaustive electronic search was performed across the Cochrane Central Register of Controlled Trials (CENTRAL), PubMed, Embase, and Epistemonikos databases. The search period spanned from January 2000 to June 2025. Search strategies were tailored to the indexing structure and syntax of each database. The query combined descriptors related to full-arch implant-supported prostheses and prosthetic materials, applying Boolean operators to maximize sensitivity and coverage. The final search strategy was as follows: ("All-on-Four" OR "Supported Fixed Complete Dental Prostheses" OR "Complete-arch implant-supported" OR "Fixed Prosthesis" OR "Fixed Dental Prostheses" OR "Full Denture" OR "Full Mouth Rehabilitation" OR "Fixed Dental Prosthesis" OR "Complete Denture") AND ("Technical Complications" OR "Prosthesis Survival Rates" OR "Metal-Acrylic" OR "Zirconia" OR "Zirconia-Based" OR "Milled Zirconia" OR "Metal Framework"). To ensure comprehensiveness, the reference lists of all included articles were manually screened for additional eligible studies. Furthermore, a complementary manual search was conducted to identify any relevant publications potentially missed by the electronic strategy. Grey literature sources were explored to minimize publication bias and ensure the broadest possible evidence base. A rigorous two-stage screening process (title/abstract and full-text review) was independently performed by three reviewers. Discrepancies were resolved through discussion or consultation with a fourth reviewer when necessary. No automation or artificial intelligence tools were employed during the selection process. For each included study, data were independently extracted regarding authorship, publication year, country of origin, outcomes evaluated, and principal conclusions. The methodological quality and risk of bias of the non-randomized studies were assessed using the Risk Of Bias In Non-randomized Studies of Interventions (ROBINS-I) tool, developed by the Cochrane Collaboration. Four authors independently performed the evaluations, and any discrepancies were resolved through consensus after structured discussion. Treatment effects were reported as relative risk (RR), odds ratio (OR), or absolute risk difference (RD) with 95% confidence intervals. RR was preferred for common outcomes, while OR was used for rare events or imbalanced groups. Meta-analyses were conducted in RevMan 5.4 using fixed- or random-effects models based on heterogeneity, quantified with I² (&lt;25% low, 25-75% moderate, &gt;75% high). Outcomes unsuitable for pooling were summarized narratively. The overall certainty of the evidence for each outcome was assessed using the Grading of Recommendations Assessment, Development and Evaluation (GRADE) methodology (7). Summary of Findings tables were constructed for principal primary outcomes following the GRADE Working Group recommendations, (Tables 1,2).

Table 1

Table 2

## Results

**Figure 1:**
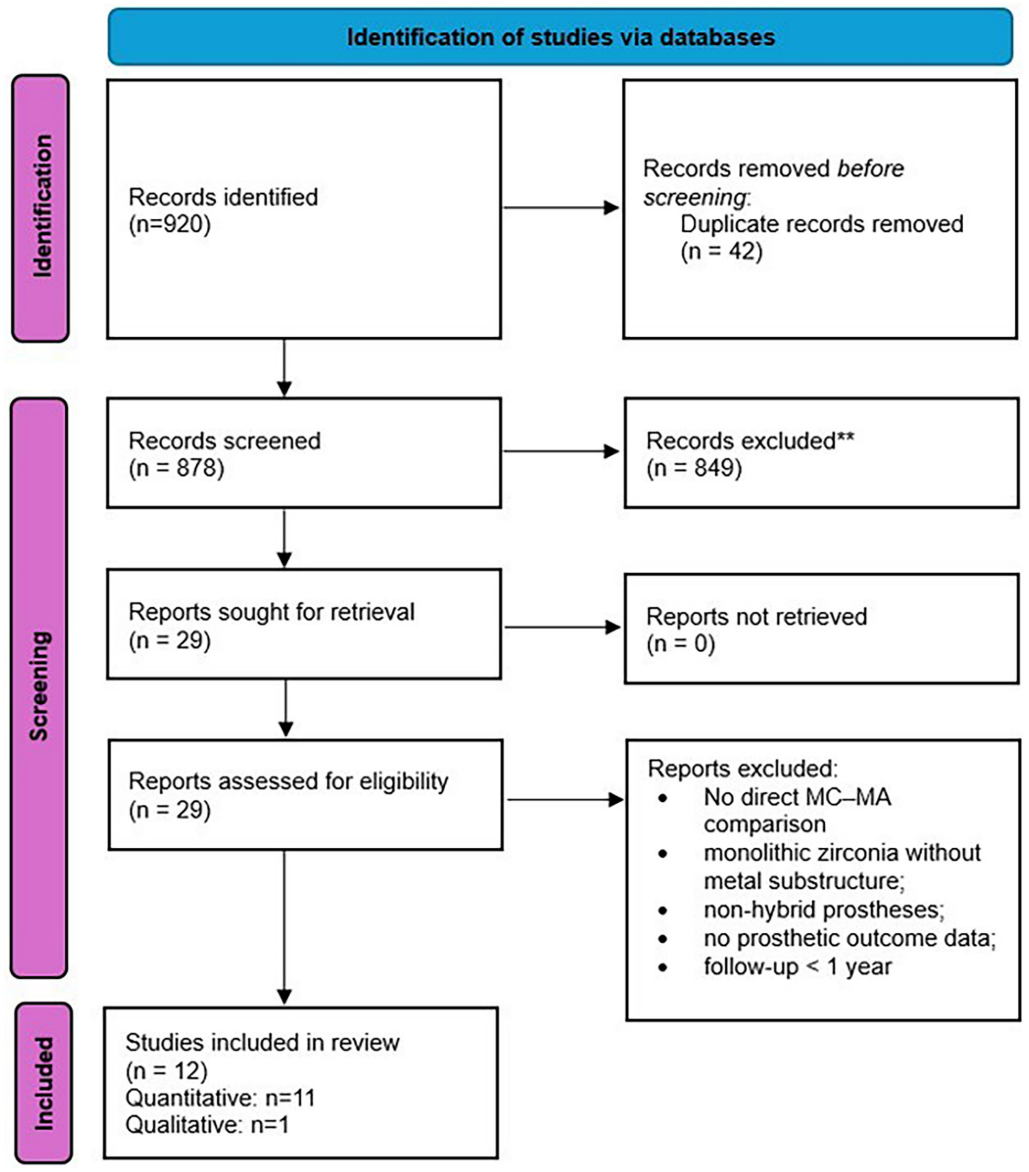
Flow diagram of identification of studies.

A total of 920 records were identified through electronic database searches. After removing 42 duplicates, 878 records were screened by title and abstract, of which 849 were excluded for not meeting the inclusion criteria, leaving 29 articles for full-text evaluation. Ultimately, 12 studies met the eligibility criteria and were included in the systematic review (8 - 19). Of these, 11 studies were incorporated into the quantitative analysis (8 - 14 , 16 - 19). The study by Peñarrocha et al. (15) was considered only for qualitative analysis, as it did not report data compatible with meta-analysis (Fig. 1).

1

**Figure 2:**
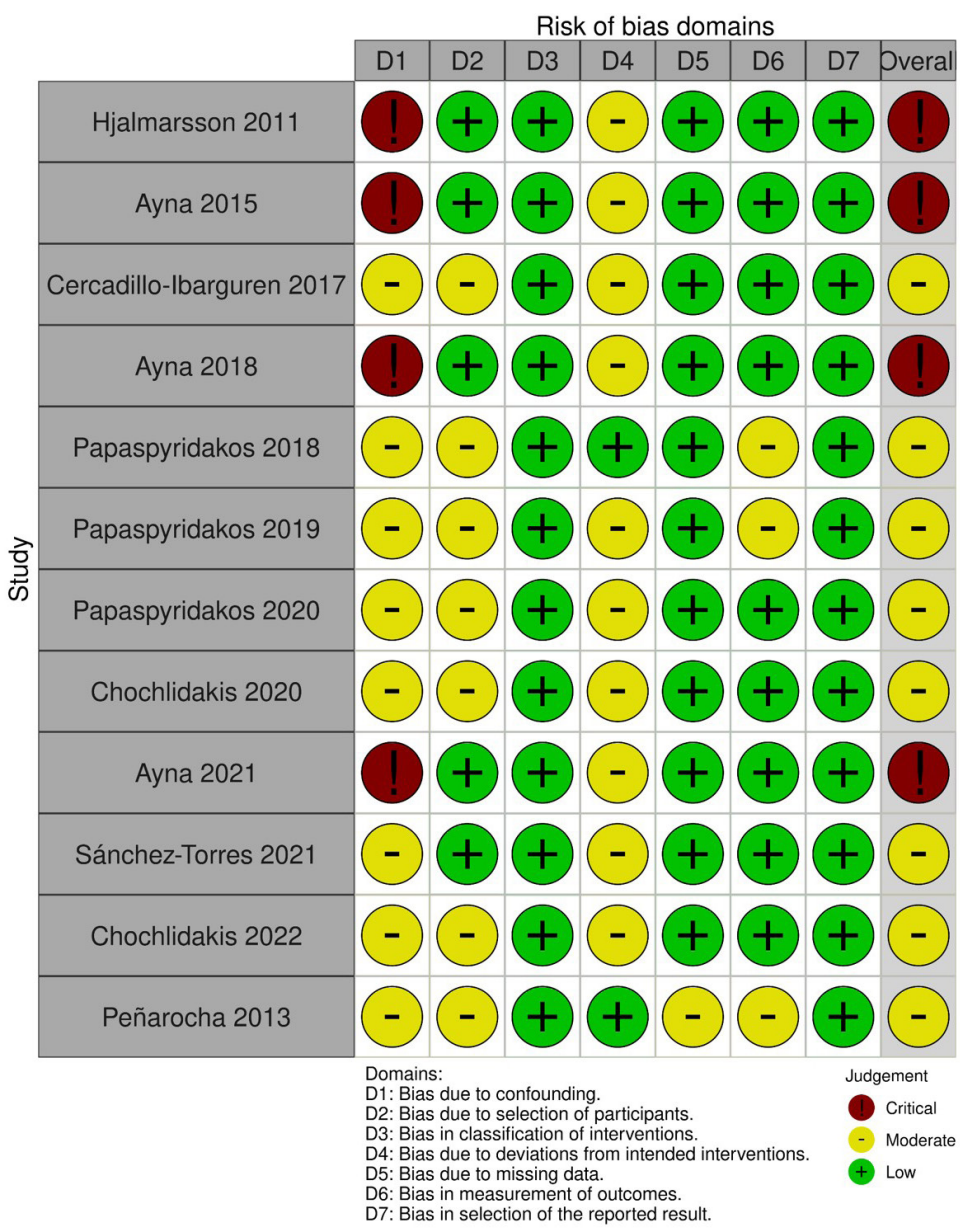
ROBINS-I bias risk plot.

Most excluded studies lacked direct comparisons between MZ and MA IS-CAPs, and several evaluated only monolithic zirconia restorations without a metallic substructure, which were beyond the scope of this review. All studies were observational, mostly retrospective, conducted across diverse countries. Participants were adults over 50 receiving full-arch fixed implant-supported prostheses with confirmed osseointegration. Interventions compared metal-zirconia and metal-acrylic prostheses on at least four implants with cast or milled frameworks and acrylic overlays. Risk of bias: Of the 12 non-randomized studies, eight showed moderate risk (12 - 17 , 19 , 20) and four critical risks (8 - 11), mainly due to confounding (D1). Participant selection (D2) and deviations from interventions (D4) were often moderate risk, while other domains including intervention classification, missing data, outcome measurement, and selective reporting (D3, D5-D7)-showed low risk (Fig. 2).

2

**Figure 3:**
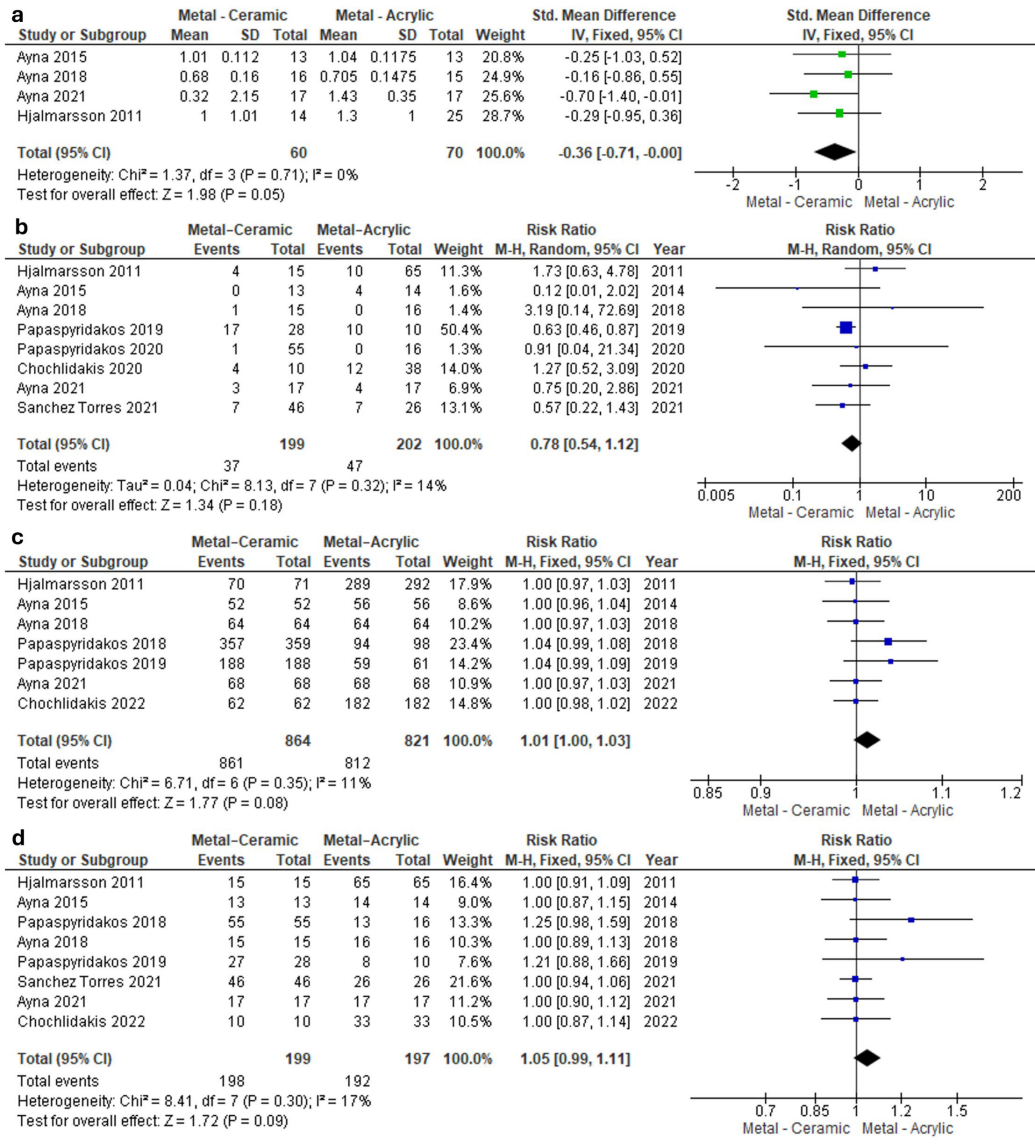
Forest plots for Outcomes 1: (a) Marginal bone loss; (b) Prosthetic tooth fracture; (c) Implant survival; (d) Prosthesis survival.

Marginal bone loss: Four non-randomized studies, (8 - 11) including 130 patients, were synthesized. The fixed-effect model demonstrated a statistically significant difference in marginal bone loss (MBL) between MZ and MA IS-CAPs, yielding an SMD of -0.36 (95% CI: -0.71 to 0.00; Z = 1.98; P = 0.05). Heterogeneity was null (I² = 0%). The certainty of evidence was rated as Moderate (Fig. 3a).

3

**Figure 4:**
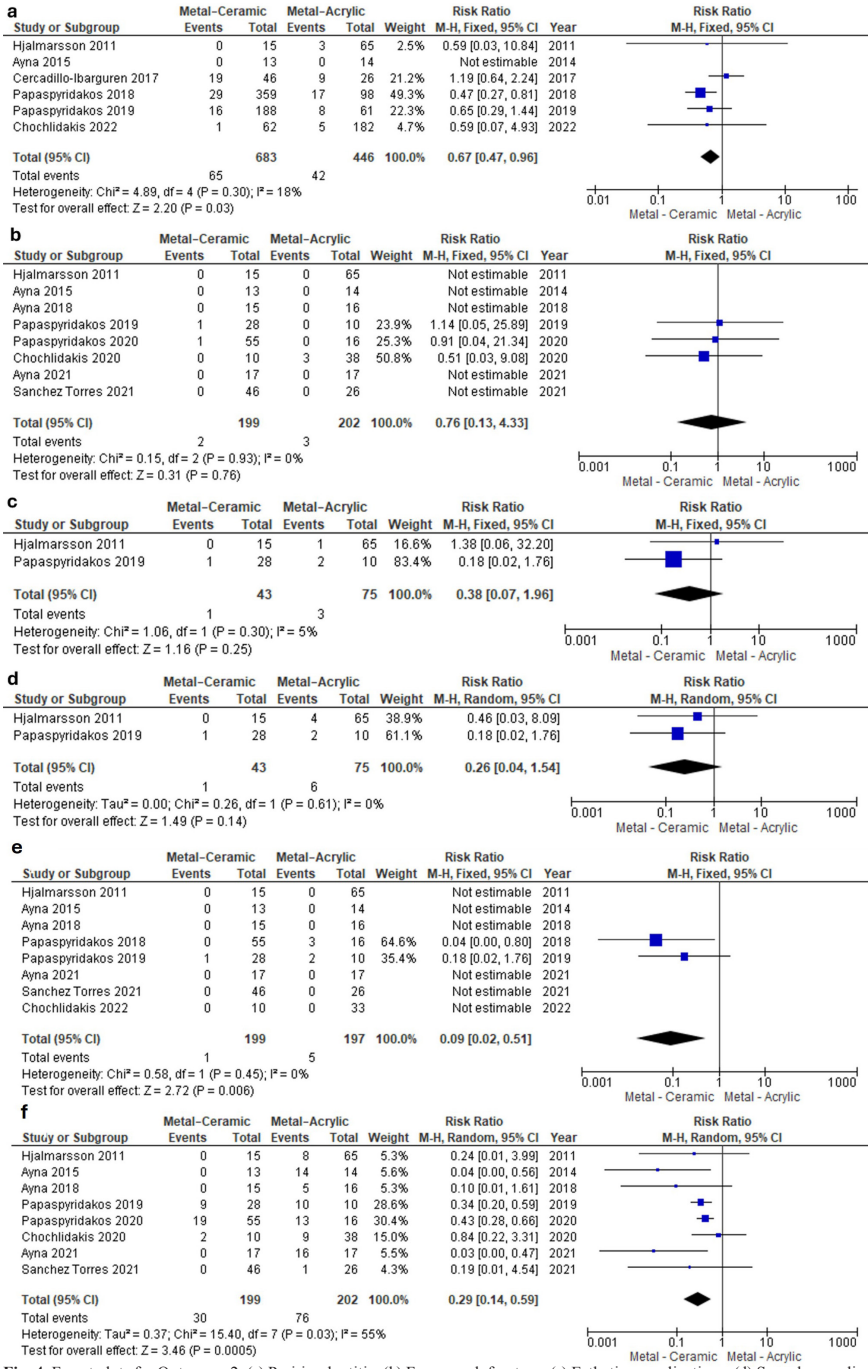
Forest plots for Outcomes 2: (a) Peri-implantitis; (b) Framework fracture; (c) Esthetic complications; (d) Speech complications.; (e) Prosthesis loss; (f) Prosthetic wear.

Prosthetic tooth fracture: Eight non-randomized studies (8 - 12 , 17 , 19 , 20), comprising 401 participants with a mean follow-up of 5 years, contributed to the pooled analysis. The fixed-effect model estimated a non-significant RR of 0.78 (95% CI: 0.54-1.12; P = 0.18), corresponding to an absolute difference of 51 fewer fractures per 1,000 MZ restorations. Heterogeneity was low (I² = 14%) (Fig. 3b). The certainty of evidence was rated as Low. Implant survival: Seven studies (8 - 11 , 13 , 16 , 17), including 1,685 implants with a mean follow-up of 5 years, were analyzed using a fixed-effects Mantel-Haenszel model. The pooled RR was 1.01 (95% CI: 1.00-1.03; Z = 1.77; P = 0.08), with an absolute difference of approximately 10 additional failures per 1,000 implants in the MZ group. Heterogeneity was low (I² = 11%) (Fig. 3c). The certainty of evidence was rated as Low. Prosthesis survival: Eight non-randomized studies (8 - 11 , 13 , 16 , 17 , 20), comprising 396 prostheses with a mean follow-up of 5 years, were synthesized. The fixed-effect model produced a pooled RR of 1.05 (95% CI: 0.99-1.11; Z = 1.72; P = 0.09). Absolute differences corresponded to 49 additional surviving prostheses per 1,000 MZ units. Heterogeneity was low (I² = 17%) (Fig. 3d). The certainty of evidence was rated as moderate. Peri-implantitis: Six non-randomized studies, (8 , 11 , 13 , 14 , 16 , 17) totaling 1,129 patients with a mean follow-up of 5 years, were included. The fixed-effect model estimated a RR of 0.67 (95% CI: 0.47-0.96; Z = 2.20; P = 0.03), equivalent to an absolute reduction of 31 peri-implantitis events per 1,000 MZ restorations. Heterogeneity was low (I² = 18%) (Fig. 4a).

4

The certainty of evidence was rated as Low. Framework fracture: Eight non-randomized studies, (8 - 12 , 17 , 19 , 20) including 401 participants with a mean follow-up of 5 years, reported five framework fracture events. The fixed-effect model yielded a non-significant RR of 0.76 (95% CI: 0.13-4.33; Z = 0.31; P = 0.76), corresponding to an absolute difference of 4 fewer events per 1,000 MZ restorations. No heterogeneity was detected (I² = 0%) (Fig. 4b). The certainty of evidence was rated as Low. Esthetic complications: Two non-randomized studies, (11 , 17) including 118 participants with a mean follow-up of 5 years, contributed four esthetic complication events. The fixed-effect model yielded a non-significant RR of 0.38 (95% CI: 0.07-1.96; Z = 1.16; P = 0.25), corresponding to 25 fewer events per 1,000 MZ restorations. Heterogeneity was negligible (I² = 5%) (Fig. 4c). The certainty of evidence was rated as Very Low. Speech complications: Two non-randomized studies, (11 , 17) totaling 118 participants with a mean follow-up of 5 years, reported seven speech-related events. The random-effects model estimated a non-significant RR of 0.26 (95% CI: 0.04-1.54; Z = 1.49; P = 0.14). The absolute difference corresponded to 59 fewer complications per 1,000 MZ restorations. No heterogeneity was observed (I² = 0%) (Fig. 4d). The certainty of evidence was rated as Very Low. Prosthesis loss: Eight non-randomized studies (8 - 11 , 13 , 16 , 17 , 20) including 396 prostheses over a mean follow-up of 5 years reported six prosthesis losses. The fixed-effect model showed a significant reduction with MZ restorations (RR = 0.09; 95% CI: 0.02-0.51; P = 0.006), corresponding to 23 fewer events per 1,000 prostheses, with no heterogeneity (I² = 0%) (Fig 4e). The certainty of evidence was Very Low. Prosthetic wear: Eight non-randomized studies, (8 , 9 - 12 , 17 , 19 , 20) totaling 401 participants with a mean follow-up of 5 years, were included. The random-effects model showed a statistically significant RR of 0.29 (95% CI: 0.14-0.59; Z = 3.46; P = 0.0005). The absolute difference corresponded to 267 fewer wear events per 1,000 MZ restorations (109 vs. 376 per 1,000). Moderate heterogeneity was observed (I² = 55%) (Fig 4f). The certainty of evidence was rated as Moderate.

## Discussion

This systematic review and meta-analysis compared monolithic zirconia IS-CAPs with a metallic substructure to conventional metal-acrylic IS-CAPs. The findings highlight how material properties and structural design shape biomechanical behavior, failure patterns, and biological performance. Mechanically, MZ prostheses are characterized by the rigidity of the zirconia-metal assembly, which limits energy absorption and channels stresses toward transitional zones, increasing susceptibility to microcracks, particularly in areas with uneven zirconia thickness or compromised bonding interfaces (21 - 24). Maintaining optimal veneer thickness (1.0-1.5 mm) and uniform support is critical to prevent chipping, the predominant failure mode in MZ prostheses (22 , 23). By contrast, MA prostheses benefit from the viscoelasticity of acrylic resin, which attenuates occlusal loads, but this advantage is offset by low fracture toughness and high wear, explaining the greater volumetric loss, tooth detachment, and prosthetic wear observed in clinical practice. These biomechanical patterns are consistent with the pooled evidence demonstrating significantly lower wear for MZ designs. Framework and connection design further influence prosthetic longevity. Internal Morse taper connections and well-integrated frameworks improve load distribution and reduce mechanical complications (23 , 25). CAD/CAM-milled or sintered frameworks offer superior dimensional precision and microstructural homogeneity compared with conventionally cast metal, minimizing residual stresses and fatigue risk (29 , 30). Digital fabrication enables controlled zirconia thickness, parallelism among implants, and more uniform stress distribution, supporting the improved resilience and reduced prosthetic wear observed in MZ prostheses. Biologically, MZ prostheses exhibited lower peri-implantitis incidence, likely due to smoother surfaces, lower porosity, reduced biofilm adhesion, and greater chemical stability relative to acrylic resin (31 , 32). These features may enhance soft-tissue compatibility and limit inflammatory responses, although further high-quality randomized studies are required to confirm these findings. Material-specific failures were observed, with MA prostheses failing mainly through wear, fracture, and tooth detachment, while MZ prostheses exhibited veneer chipping or delamination due to zirconia brittleness and stress concentration. These patterns highlight the importance of careful prosthetic design, adequate load distribution, and optimal veneer support to maximize long-term outcomes. The antagonist arch may play a clinically relevant role in the development of mechanical and biological complications. Differences in occlusal scheme, material properties, and force distribution between natural dentition, complete dentures, and implant-supported fixed prostheses could partially explain the heterogeneity observed among studies. Unfortunately, the limited and inconsistent reporting of antagonist characteristics prevented formal assessment of this variable in the present analysis. A key limitation of this review is its reliance on predominantly non-randomized and retrospective studies, many of which presented serious or critical risk of bias according to the ROBINS-I tool. Most included investigations were subject to confounding, selection bias, and heterogeneity in prosthetic designs, occlusal protocols, and follow-up periods, which limits generalizability. Consequently, the overall certainty of evidence, as assessed by the GRADE approach, ranged from moderate to very low for several outcomes, including marginal bone loss, implant survival, and peri-implantitis. These limitations require cautious interpretation of the pooled estimates and preclude definitive causal inferences. Therefore, the conclusions of this meta-analysis should be considered preliminary. High-quality randomized controlled trials with standardized protocols and long-term follow-up are needed to confirm these findings.

## Conclusions

Within the limitations of mainly non-randomized evidence, monolithic zirconia IS-CAPs with a metallic substructure demonstrated a more favorable clinical profile than metal-acrylic prostheses, with lower prosthetic wear and reduced peri-implantitis. Trends also favored improved prosthesis survival and reduced loss, while marginal bone loss was lower for MZ prostheses. Although veneer chipping remains a material-specific limitation, overall complications were predictable. These findings support MZ hybrids as a durable and biologically advantageous option, but the predominance of observational studies and low certainty of evidence underscore the need for high-quality randomized trials to confirm these results. Clinical Significance Metal-zirconia implant-supported screw-retained complete-arch prostheses may reduce prosthetic wear, peri-implantitis, and marginal bone loss compared with conventional metal-acrylic designs. These findings support their use as a predictable alternative for full-arch implant rehabilitation and may assist clinicians in balancing long-term biological stability, mechanical performance, and material selection.

## Figures and Tables

**Table 1:** Characteristics of included studies.

Study (Year)	Country	Study design	Prostheses(number)	Intervention	Follow up (years)	Principal conclusions
Hjalmarsson 2011	Sweden	Retrospective observational	80	Prostheses MZ: 15Prostheses MA: 65	5	Metal–zirconia and metal–acrylic prostheses demonstrated comparable clinical performance in terms of bone stability and peri-implant health.
Ayna 2014	Germany	Retrospective observational	27	Prostheses MZ: 14Prostheses MA: 13	5	Both types of prostheses yielded similar outcomes in terms of bone loss and patient satisfaction; however, metal–zirconia restorations showed less peri-implant inflammation, greater occlusal strength, and an absence of prosthetic complications compared to the acrylic group. These findings suggest greater long-term clinical predictability for the zirconia option.
Cercadillo-Ibarguren 2017	Spain	Retrospective observational	72	Prostheses MZ: 46Prostheses MA: 26	4	No significant clinical differences were observed between metal–zirconia and metal–acrylic prostheses regarding peri-implant inflammation or the rate of technical complications. Both groups showed favorable survival outcomes but a high prevalence of peri-implant diseases, underscoring the importance of regular maintenance visits regardless of the material used.
Ayna 2018	Germany	Retrospective observational	31	Prostheses MZ: 15Prostheses MA: 16	7	Both prosthetic options provided similar outcomes in terms of bone loss and patient satisfaction. However, metal–zirconia prostheses demonstrated better peri-implant health, whereas acrylic restorations—despite being associated with greater plaque accumulation and bleeding—were easier and more cost-effective to repair.
Papaspyridakos 2018	USA	Retrospective observational	71	Prostheses MZ: 55Prostheses MA: 16	5	Although both prosthesis types exhibited similar survival rates, metal–zirconia prostheses showed fewer technical complications and required less maintenance compared with metal–acrylic ones. This suggests greater long-term clinical stability for zirconia restorations, making them a more favorable option in terms of durability despite their higher initial cost.
Papaspyridakos 2019	USA	Retrospective observational	38	Prostheses MZ: 28Prostheses MA: 10	5	Both prosthesis types achieved high survival rates for both implants and frameworks. However, metal–acrylic prostheses exhibited a higher frequency of technical complications and accounted for all prosthetic failures reported in the study. Although no significant differences were observed in biological complications, the findings suggest that metal–zirconia prostheses provide greater clinical durability against mechanical failures.
Papaspyridakos 2020	USA	Retrospective observational	71	Prostheses MZ: 55Prostheses MA: 16	5	Metal–acrylic frameworks exhibited significantly more technical complications and a higher rate of prosthetic failures. Metal–zirconia prostheses showed less wear, fewer fractures, and greater patient satisfaction, establishing themselves as the option with superior long-term clinical performance. These differences were statistically significant, particularly regarding major complications and durability.
Chochlidakis 2020	USA	Retrospective observational	48	Prostheses MZ: 10Prostheses MA: 38	3	Metal–acrylic and metal–zirconia prostheses showed similar survival and complication rates. No significant differences were observed in clinical performance or patient satisfaction, suggesting that both types of prostheses are viable alternatives with comparable complication profiles.
Chochlidakis 2022	USA	Retrospective observational	43	Prostheses MZ: 10Prostheses MA: 33	5	Both prosthesis types achieved a 100% survival rate; however, metal–acrylic prostheses were associated with a higher incidence of biological complications, including soft tissue hypertrophy and greater plaque accumulation, compared with metal–zirconia prostheses. Although mucositis and peri-implantitis were common in both groups, the findings suggest that the prosthetic material significantly influences peri-implant health, with the zirconia option being less prone to these complications.
Ayna 2021	Germany	Retrospective observational	34	Prostheses MZ: 17Prostheses MA: 17	6	Both superstructures provided satisfactory long-term outcomes; however, metal–zirconia prostheses exhibited less marginal bone loss, reduced plaque accumulation, and lower bleeding on probing. Although both options were functional and well accepted by patients, the clinical findings favored the zirconia prostheses as the option with superior biological behavior and a lower risk of peri-implant inflammation.
Peñarrocha 2013	Spain	Retrospective observational	50	Prostheses MZ: 25Prostheses MA: 25	5	No statistically significant differences were observed between the groups; however, metal–zirconia prostheses showed slightly better satisfaction outcomes, possibly related to more favorable initial anatomical conditions. Both types of rehabilitation proved functional and showed a low incidence of complications, supporting their viability in atrophic maxillar.Metal–acrylic and metal–zirconia prostheses demonstrated comparable clinical outcomes, with high survival rates and a low incidence of complications. No significant differences were observed between the groups in terms of peri-implant health, mechanical failures, or patient satisfaction, suggesting that both options are clinically viable in the long term.
Sánchez Torres 2021	Spain	Retrospective observational	33	Prostheses MZ: 15Prostheses MA: 18	4	Metal–acrylic and metal–zirconia prostheses demonstrated comparable clinical outcomes, with high survival rates and a low incidence of complications. No significant differences were observed between the groups in terms of peri-implant health, mechanical failures, or patient satisfaction, suggesting that both options are clinically viable in the long term.

1

**Table 2:** Summary of Findings for Metal - Zirconia Versus Metal–Acrylic for Implant-supported screw-retained complete-arch prosthesis.

Outcome and follow-up	Patients (studies), N	Relative effect(95% CI)	Absolute effects (95% CI)			Certainty
			Metal–Acrylic	Metal - Zirconia	Difference	
Marginal bone loss, mmFollow-up: mean 5 years	130(4 non-randomised studies)	-	-	-	-0.36(-0.71 to 0)	ѲѲѲΟ Moderate
Prosthetic tooth fractureFollow-up: mean 5 years	401(8 non-randomised studies)	RR = 0.78(0.54 to 1.12)	233 per 1000	181 per 1000(126 to 261)	51 fewer per 1000(from 107 fewer to 28 more)	ѲѲѲΟ Moderate
Implant survivalFollow-up: mean 5 years	1685(7 non-randomised studies)	RR = 1.01(1.00 to 1.03)	989 per 1000	999 per 1000(989 to 1000)	10 more per 1000(from 0 fewer to 30 more)	ѲѲΟΟ Low
Prosthesis survivalFollow-up: mean 5 years	396(8 non-randomised studies)	RR = 1.05(0.99 to 1.11)	975 per 1000	1000 per 1000(965 to 1000)	49 more per 1000(from 10 fewer to 107 more)	ѲѲѲΟ Moderate
Peri-implantitisFollow-up: mean 5 years	1129(6 non-randomised studies)	RR = 0.67(0.47 to 0.96)	94 per 1000	63 per 1000(44 to 90)	31 fewer per 1000(from 50 fewer to 4 fewer)	ѲѲΟΟ Low
Framework fractureFollow-up: mean 5 years	401(8 non-randomised studies)	RR = 0.76(0.13 to 4.33)	15 per 1000	11 per 1000(2 to 64)	4 fewer per 1000(from 13 fewer to 49 more)	ѲѲΟΟ Low
Esthetic complicationsFollow-up: mean 5 years	118(2 non-randomised studies)	RR = 0.38(0.07 to 1.96)	40 per 1000	15 per 1000(3 to 78)	25 fewer per 1000(from 37 fewer to 38 more)	ѲѲΟΟ Very low
Speech complicationsFollow-up: mean 5 years	118(2 non-randomised studies)	RR = 0.26(0.04 to 1.54)	80 per 1000	21 per 1000(3 to 123)	59 fewer per 1000(from 77 fewer to 43 more)	ѲΟΟΟ Very low
Prosthesis lossFollow-up: mean 5 years	396(8 non-randomised studies)	RR = 0.09(0.02 to 0.51)	25 per 1000	2 per 1000(1 to 13)	23 fewer per 1000(from 25 fewer to 12 fewer)	ѲΟΟΟ Very low
Prosthetic wearFollow-up: mean 5 years	401(8 non-randomised studies)	RR = 0.29(0.14 to 0.59)	376 per 1000	109 per 1000(53 to 222)	267 fewer per 1000(from 324 fewer to 154 fewer)	ѲΟΟΟ Moderate

2

## Data Availability

The datasets used and/or analyzed during the current study are available from the corresponding author.

## References

[1] Mac Giolla Phadraig C, Nunn J, McCallion P, McCarron M (2018). Prevalence of edentulism among adults with intellectual disabilities: a narrative review informed by systematic review principles. Spec Care Dentist.

[2] Egilmez F, Ergun G, Cekic-Nagas I, Bozkaya S (2015). Implant-supported hybrid prosthesis: conventional treatment method for borderline cases. Eur J Dent.

[3] Bhering CLB, Mesquita MF, Kemmoku DT, Noritomi PY, Consani RLX, Barao VAR (2016). Comparison between All-on-four and All-on-six treatment concepts and framework material on stress distribution in atrophic maxilla: a prototyping-guided 3D finite element analysis study. Mater Sci Eng C Mater Biol Appl.

[4] Pjetursson BE, Thoma D, Jung R, Zwahlen M, Zembic A (2012). A systematic review of the survival and complication rates of implant-supported fixed dental prostheses after a mean observation period of at least 5 years. Clin Oral Implants Res.

[5] Fischer K, Stenberg T (2013). Prospective 10-year cohort study based on a randomized controlled trial on implant-supported full-arch maxillary prostheses. Part II: prosthetic outcomes and maintenance. Clin Implant Dent Relat Res.

[6] Liberati A, Altman DG, Tetzlaff J (2009). The PRISMA statement for reporting systematic reviews and meta-analyses of studies that evaluate health care interventions: explanation and elaboration. J Clin Epidemiol.

[7] Guyatt GH, Oxman AD, Santesso N (2013). GRADE guidelines: 12. Preparing summary of findings tables—binary outcomes. J Clin Epidemiol.

[8] Ayna M, Gulses A, Acil Y (2015). Comprehensive comparison of the 5-year results of All-on-4 mandibular implant systems with acrylic and zirconia suprastructures. J Oral Implantol.

[9] Ayna M, Karayurek F, Jepsen S (2021). Six-year clinical outcomes of implant-supported acrylic vs zirconia superstructures according to the All-on-4 treatment concept for the rehabilitation of the edentulous maxilla. Odontology.

[10] Ayna M, Gulses A, Acil Y (2018). A comparative study on 7-year results of the All-on-4 immediate-function concept for completely edentulous mandibles: metal-zirconia vs bar-retained superstructures. Odontology.

[11] Hjalmarsson L, Smedberg JI, Pettersson M, Jemt T (2011). Implant-level prostheses in the edentulous maxilla: a comparison with conventional abutment-level prostheses after 5 years of use. Int J Prosthodont.

[12] Papaspyridakos P, Bordin TB, Kim YJ (2020). Technical complications and prosthesis survival rates with implant-supported fixed complete dental prostheses: a retrospective study with 1- to 12-year follow-up. J Prosthodont.

[13] Chochlidakis K, Ercoli C, Einarsdottir E (2022). Implant survival and biologic complications of implant fixed complete dental prostheses: an up to 5-year retrospective study. J Prosthet Dent.

[14] Cercadillo-Ibarguren I, Sanchez-Torres A, Figueiredo R, Schwarz F, Gay-Escoda C, Valmaseda-Castellon E (2017). Immediately loaded implant-supported full-arches: peri-implant status after 1-9 years in a private practice. J Dent.

[15] Penarrocha-Oltra D, Candel-Marti E, Penarrocha-Diago M, Martinez-Gonzalez JM, Aragoneses JM, Penarrocha-Diago M (2013). Palatal positioning of implants in severely atrophic edentulous maxillae: five-year cross-sectional retrospective follow-up study. Int J Oral Maxillofac Implants.

[16] Papaspyridakos P, Bordin TB, Kim YJ (2018). Implant survival rates and biologic complications with implant-supported fixed complete dental prostheses: a retrospective study with up to 12-year follow-up. Clin Oral Implants Res.

[17] Papaspyridakos P, Bordin TB, Natto ZS (2019). Double full-arch fixed implant-supported prostheses: outcomes and complications after a mean follow-up of 5 years. J Prosthodont.

[19] Chochlidakis K, Einarsdottir E, Tsigarida A (2020). Survival rates and prosthetic complications of implant fixed complete dental prostheses: an up to 5-year retrospective study. J Prosthet Dent.

[20] Sanchez-Torres A, Cercadillo-Ibarguren I, Figueiredo R, Gay-Escoda C, Valmaseda-Castellon E (2021). Mechanical complications of implant-supported complete-arch restorations and impact on patient quality of life: a retrospective cohort study. J Prosthet Dent.

[21] Rodriguez LC, Saba JN, Meyer CA, Chung KH, Wadhwani C, Rodrigues DC (2016). A finite element analysis of novel vented dental abutment geometries for cement-retained crown restorations. Clin Exp Dent Res.

[22] Jerman E, Lumkemann N, Eichberger M (2021). Evaluation of translucency, Martens hardness, biaxial flexural strength and fracture toughness of 3Y-TZP, 4Y-TZP and 5Y-TZP materials. Dent Mater.

[23] AlTarawneh S, Thalji G, Cooper L (2021). Full-arch implant-supported monolithic zirconia fixed dental prostheses: an updated systematic review. Int J Oral Implantol.

[24] Happe A, Roling N, Schafer A, Rothamel D (2015). Effects of different polishing protocols on the surface roughness of Y-TZP surfaces used for custom-made implant abutments: a controlled morphologic SEM and profilometric pilot study. J Prosthet Dent.

[25] Fuda S, Martins BGDS, Castro FC (2023). Marginal bone level and clinical parameter analysis comparing external hexagon and Morse taper implants: a systematic review and meta-analysis. Diagnostics.

[26] Chen X, Xu Z, Gai K, Pei X, Li R, Wan Q (2025). Biomechanical analysis of All-on-4 implant-supported framework using different materials across various clinical practice. BMC Oral Health.

[27] Katsoulis J, Takeichi T, Sol Gaviria A, Peter L, Katsoulis K (2017). Misfit of implant prostheses and its impact on clinical outcomes: definition, assessment and a systematic review of the literature. Eur J Oral Implantol.

[28] Buzayan MM, Yunus NB (2014). Passive fit in screw-retained multi-unit implant prosthesis: understanding and achieving. J Indian Prosthodont Soc.

[29] Sahin Hazir D, Sozen Yanik I, Guncu MB, Canay RS (2025). Biomechanical behavior of titanium, cobalt-chromium, zirconia, and PEEK frameworks in implant-supported prostheses: a dynamic finite element analysis. BMC Oral Health.

[30] Padros R, Punset M, Molmeneu M (2020). Mechanical properties of CoCr dental-prosthesis restorations made by three manufacturing processes: influence of the microstructure and topography. Metals.

[31] Egawa M, Miura T, Kato T, Saito A, Yoshinari M (2013). In vitro adherence of periodontopathic bacteria to zirconia and titanium surfaces. Dent Mater J.

[32] Sadid-Zadeh R, Willis J, Forgo G, Haraszthy V (2020). Comparative analysis of biofilm formation on materials used for the fabrication of implant-supported prostheses. Braz Dent J.

